# A clustering approach to identify multidimensional poverty indicators for the bottom 40 percent group

**DOI:** 10.1371/journal.pone.0255312

**Published:** 2021-08-02

**Authors:** Mariah Abdul Rahman, Nor Samsiah Sani, Rusnita Hamdan, Zulaiha Ali Othman, Azuraliza Abu Bakar

**Affiliations:** Center for Artificial Intelligence Technology, Faculty of Information Science & Technology, Universiti Kebangsaan Malaysia, Bangi, Selangor, Malaysia; Universidad Nacional Autonoma de Nicaragua Leon, NICARAGUA

## Abstract

The Multidimensional Poverty Index (MPI) is an income-based poverty index which measures multiple deprivations alongside other relevant factors to determine and classify poverty. The implementation of a reliable MPI is one of the significant efforts by the Malaysian government to improve measures in alleviating poverty, in line with the recent policy for Bottom 40 Percent (B40) group. However, using this measurement, only 0.86% of Malaysians are regarded as multidimensionally poor, and this measurement was claimed to be irrelevant for Malaysia as a country that has rapid economic development. Therefore, this study proposes a B40 clustering-based K-Means with cosine similarity architecture to identify the right indicators and dimensions that will provide data driven MPI measurement. In order to evaluate the approach, this study conducted extensive experiments on the Malaysian Census dataset. A series of data preprocessing steps were implemented, including data integration, attribute generation, data filtering, data cleaning, data transformation and attribute selection. The clustering model produced eight clusters of B40 group. The study included a comprehensive clustering analysis to meaningfully understand each of the clusters. The analysis discovered seven indicators of multidimensional poverty from three dimensions encompassing education, living standard and employment. Out of the seven indicators, this study proposed six indicators to be added to the current MPI to establish a more meaningful scenario of the current poverty trend in Malaysia. The outcomes from this study may help the government in properly identifying the B40 group who suffers from financial burden, which could have been currently misclassified.

## Introduction

Malaysia has experienced significant progress in poverty reduction over half a century ago with tremendous initiatives made by the government since the introduction of the New Economic Policy (NEP) in 1971 [1]. Afterwards, the New Economic Model (NEM) was launched in 2010 with the main objective to make Malaysia a high-income and developed country by 2020. As such, the National Economic Advisory Council (MPEN) had suggested that the B40 group who are less fortunate and needs special attention should be focused on [2]. In regard to this, in the 10th Malaysia Plan (10MP) in 2011, the government took various efforts to increase the income of this group [3]. Later, in the 11th Malaysia Plan (11MP), the government continued its intensive efforts to support the development of the B40 group, which includes addressing issues regarding cost of living and strengthening the mechanism of assistance [4]. Likewise, through the 2019 Budget, which was unveiled in November 2018, the government committed to continuing and improving the Cost of Living Aid to the 2.7 million B40 group by providing a more targeted assistance. Health insurance and medical protection were also provided through the National Health Protection Fund, besides introducing the Healthcare Protection Scheme [5].

Poverty Line Income (PLI) is an income approach in one dimension, specifically measuring the gross monthly household income. Thus, the main weakness of such approach is that it does not represent an accurate and complete picture of deprivation and human well-being. The approach also gauges only the minimum requirement for basic needs and living standard, which does not consider the households’ preferences and does not reflect social mobility in the society. The PLI misrepresents what is available to a household for the purpose of meeting its basic needs. A family’s living conditions are shaped by more than the current income, and households may experience different living standards for reasons not explained by their current income data. This can also be regarded as a consumption bias, focusing less on human capability and potential. Generally, Malaysians are classified into three categories of income groups based on the household income: the top 20 percent of Malaysian population (T20), the middle 40 percent (M40) and the bottom 40 percent (B40). Table 1 shows the income classification based on the findings in 2016 and 2019 Household Income and Basic Amenities Survey. This study used the 2016 income threshold. T20 households earned over RM 9,620 per month, M40 households earned between RM 4,360 and RM 9,619 per month, and B40 households earned lesser than RM 4,360 per month.

**Table 1:** Income classification for Malaysia.

Term	Description	Monthly Income Threshold	
		2016	2020
T20	Top 20 percent	≥9,620	≥10,960
M40	Middle 40 percent	4,360–9,619	4,850–10,959
B40	Bottom 40 percent	<4,360	<4,850

Source: Household income and basic amenities survey

At present, 2.78 million households earning a monthly income less than RM 4,360 are categorized as B40 in Malaysia. From this figure, three subgroups of B40 are identified, in which 24.1% of them are from lower-middle income category, 15.5% from low income, and 0.4% are categorized as poor [6]. Each subgroup represents different characteristics and needs. Thus, in order to improve the well-being of different subgroups of B40, the interpretations of poverty that should be viewed from various dimensions, in order to reflect the actual state of poverty.

On July 2010, the Oxford Poverty and Human Development Initiative (OPHI) and the United Nations Development Programme (UNDP) proposed a new poverty measure. They introduced the Multidimensional Poverty Index (MPI), which complements traditional income-based poverty indices by measuring multiple dimensions and different factors to determine and classify poverty. Based on the global MPI 2018, there are 3 dimensions namely health, education and living standards comprising 10 indicators namely nutrition, child mortality, years of schooling, school attendance, cooking fuel, sanitation, drinking water, electricity, housing and assets. Each dimension has the same weight as one third. The MPI looks at poverty from a surpassing perspective and sees how poverty can be experienced in many ways at the same time. The multidimensional measures satisfy several useful properties which allow, for instance, poverty targeting and comparisons over time and across countries and regions. In accordance with that, Malaysia has also taken steps to develop its custom Multidimensional Poverty Index (MPI) model at the national level as outlined in the Eleventh Malaysia Plan (11MP), following the footsteps of 100 countries worldwide that have already adopted the methods launched by OPHI in 2010 [7]. It also complements the PLI by considering other aspects apart from income.

Malaysian MPI covered four dimensions: education, health, living standards and income with 11 indicators: schooling years, school attendance, healthcare access, clean water access, living place conditions, room crowdedness, toilet, garbage collection facility, transportation, basic communication tools and mean monthly household income [4]. However, according to a recent mid-term review of the 11th Malaysia Plan released on October 2018, the index calculated using the MPI model was reported to be at 0.0033 while the incidence of multidimensional poverty was 0.86% at the national level for 2016 [6]. According to Dr Kenneth Simler, a Senior Economist of World Bank Group Global Knowledge and Research Hub Malaysia, the index is too low for Malaysia and it was recommended to increase the benchmark or the so-called deprivation cut-off level by using both MPI and PLI model in the future [8]. The multidimensional measures satisfy several useful properties which allow, for instance, poverty targeting and comparisons over time and across countries and regions. However, it is crucial to identify the indicators that are important for the MPI classification, which can be used by the government for further strategic planning in response to the poverty elimination. The recognition of these limitations has led us to propose this study in using data analytics approach to identify relevant indicators for multidimensional poverty classification. The proposed study makes use of clustering machine learning for poverty classification.

Machine learning methods are the most commonly used methods for predicting poverty. There are two main groups in machine learning methods, namely, supervised and unsupervised learning. Supervised learning is one of the ways in which the learning environment (also known as training data which contains user-defined labels) is formed and delivered. The algorithm will repeat the predictions using training data, and the learning will stop once it has achieved a certain level of performance. Then, a test set is performed to verify the accuracy of the predictions. In contrast, in unsupervised learning, the data on learning process is unlabeled to view unusual structures or patterns without clear learning goals [9–11]. Many studies have been conducted in analyzing multidimensional poverty using machine learning methods such as classification and clustering [12–17]. Clustering technique is a method of collecting data objects and grouping them based on the similarity of objects to gain an in-depth understanding of data distribution. In general, there are five key approaches to clustering, namely partitioning, hierarchy, density-based, grid-based and model-based [18].

To date, many studies have been published in the B40 domain. Mohd Zain and Tambi described the B40 group as urban poor in Malaysia and studied the factor of urban poverty in the development of late bloomer in education [19]. Whereas, Abdullah and Mohammad studied the health and literacy level among B40 and M40 men and demographic factors related to health literacy [20]. On the other hand, a group of researchers looked at the causes contributing to the increasing cost of living in this group [21]. Studies by Mayan, Mohd Nor and Samat examined the challenges faced in increasing the income of the B40 group [22]. A recent study conducted by Sani has classified the B40 group by a predictive model using the machine learning method. The researchers compared the performance of the three classification algorithms namely the Naïve Bayes, Decision Tree and k-Nearest Neighbor (kNN) and concluded that the Decision Tree model is the best model for classifying the B40 group [9].

In the past few decades, many researchers have developed a large number of clustering algorithms such as partitional, hierarchical and density-based clustering (DBC) methods. Those clustering algorithms have been applied in a wide variety of domain, such as image processing, data mining, market segmentation, medical imaging, social networks and including poverty. For instance, Ahmad and Ejaz [23] used the Two-Step Cluster Analysis technique. They found out that the ratio of sex, income and education were the crucial contributing factors in the non-poor group while dependence rate and family size were the crucial contributing factors in the poor group. Apart from that, the Analytic Hierarchy Process (AHP) was applied for poverty classification, while K-Means clustering was used to determine the range values between clusters [24]. Likewise, Coromaldi and Drago [25] employed the K-Means algorithm to explain poverty in Italy through an in-depth study of the income-deprivation score relationship. Their research found that poverty analysis is strengthened by examining the relationship between income and deprivation score using the multidimensional poverty indicators. On top of that, Chamboko and Re [17] have mapped multiple deprivation patterns for 13 areas in Namibia using GIS application and using the K-Means algorithm for clustering purposes. To build scores and thus reduce the number of deprivation dimensions, they applied Principal Component Analysis (PCA). This study looks at the relationship between deprivation and demographic characteristics based on the clusters produced.

Another research relevant to poverty using machine learning was done by Santoso and Irawan [26] using K-nearest neighbor (k-NN) and learning vector quantization (LVQ). In their research, K-NN produced higher accuracy as compared to LVQ. Similarly, Sano and Nindito [27] from Indonesia used K-Means algorithm for clustering the poverty. More interesting research was carried out by Njuguna and McSharry [28], who constructed spatiotemporal poverty indices through mobile telephone activity as an alternative to classify poverty using linear regression. Based on the research conducted thus far, there is a huge opportunity to discover a machine learning technique to classify multidimensional poverty according to the Malaysian context. The capability of machine learning in dealing with a large amount of data that can reveal data pattern may contribute to a higher accuracy of a poverty prediction model [29, 30].

In summary, from the above study, it can be concluded that there is a need for a comprehensive study on the measurement of multidimensional poverty to improve the current national MPI. Therefore, in this work, we have identified that there is a great opportunity to develop a clustering model that can identify Multidimensional Poverty Indicators and dimensions for the B40 group in Malaysia. After considering a number of well-known clustering algorithm, the K-Means algorithm is suggested in this study. The contributions of this paper are summarized below:

- Proposed B40 clustering-based K-Means architecture to identify the right indicators and dimensions that yield more precise MPI measurement.
- Extensive clustering analysis identified seven indicators of multidimensional poverty among B40 group. Out of the seven, six indicators (i.e. literacy, highest education level and grade, housing, access to television services, assets, and work) from three dimensions (i.e. education, living standard and employment) are proposed to be added to the current national MPI.

- Employment is identified as an additional dimension for the consideration of policymakers towards MPI establishment.
- The relevant indicators and dimensions are required and can guide the government in formulating an MPI to ensure the needs of B40 group are adequately addressed
- Outcomes from this study help government to efficiently identify B40 group, which otherwise could be misclassified.

## Research methodology

**Fig 1:**
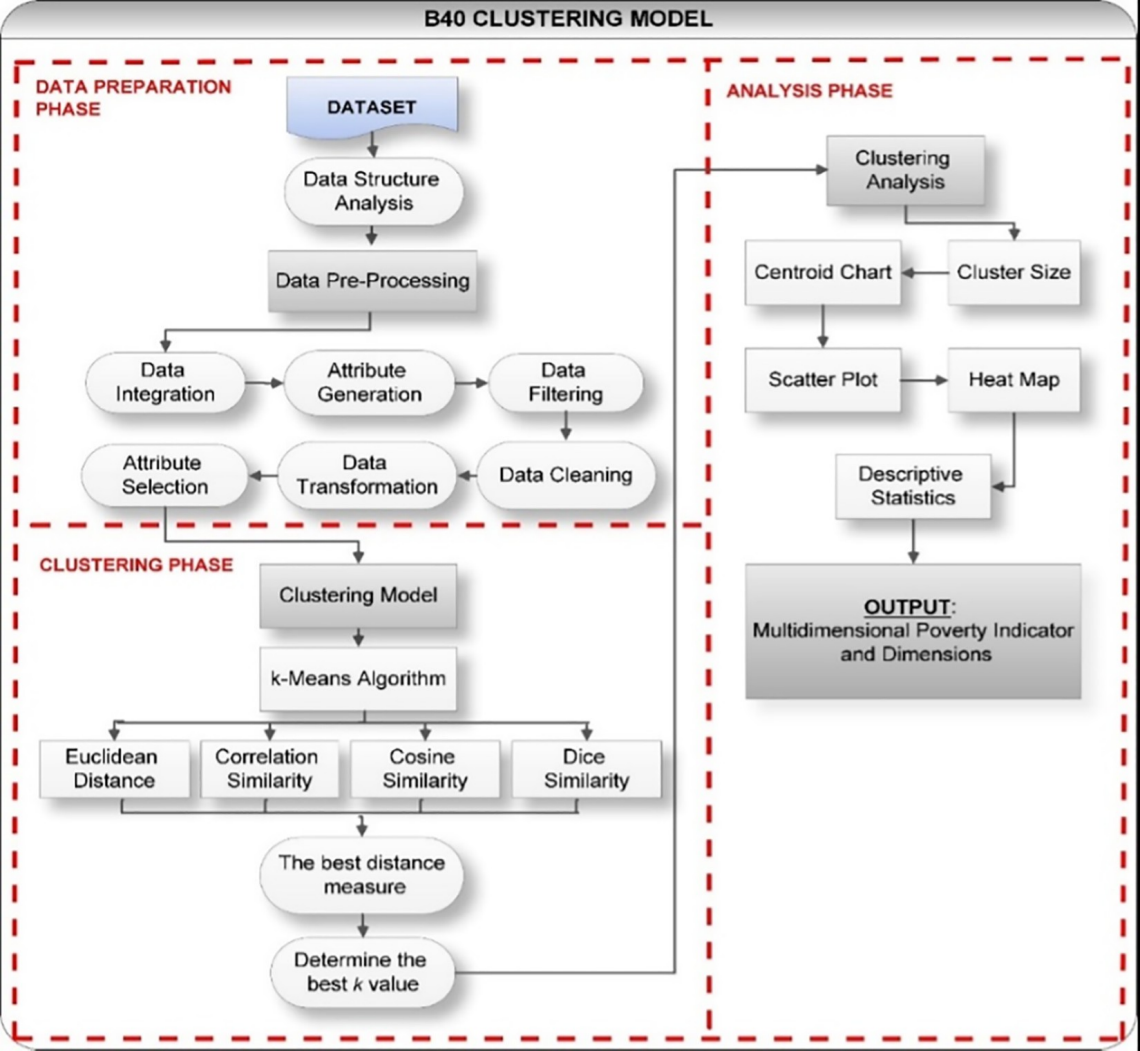
The workflow for the B40 clustering model.

The overall architecture of the proposed method for identifying key indicators of multidimensional poverty among B40 group is depicted in Fig 1. The workflow comprises three main phases, namely data preparation phase, clustering phase and analysis phase, as shown in Fig 1. The data preparation phase starts with analyzing the structured data collected by the Malaysian Department of Statistics (DOSM), from the Malaysian Population and Housing Census 2010, consisting of 532,298 households. The 2010 Population and Housing Census of Malaysia [31] was the fifth decennial census to be conducted since the formation of Malaysia in 1963. The previous censuses were conducted in 1970, 1980, 1991 and 2000, indicating that each census was conducted once every decade. Census is an enormous statistical project that has been carried out in order to produce very useful data for planning and implementation of national development. The data collected will provide a comprehensive set of information on population, various demographic, social and economic features. Furthermore, the census data provides information on the total stock of residence, basic amenities and housing requirements available.

The raw dataset would go through data pre-processing phase before clustering phase takes place. In clustering phase, K-Means algorithm was tested with four different distance measure: Euclidean Distance (ED), Correlation Similarity (CrS), Cosine Similarity (CS) and Dice Similarity (DS) to choose the best distance measure. Then, experiments were conducted and evaluated from *k* value equals to 2 up to 15 in order to determine the best *k*. Finally, a series of analysis was performed by looking at the cluster size, centroid chart, scatter plot analysis, heat map analysis, and descriptive statistics method to investigate the pattern of each cluster formed further. The data preprocessing and experiments are conducted using Rapidminer Studio tools.

### Data preprocessing

Data preprocessing methods focus on altering the raw data in an effort to assess the consistency of the data that satisfies the clustering process criteria. In this phase, six pre-processing activities are involved as depicted in Fig 1, namely data integration, attribute generation, data filtering, data cleaning, data transformation, and attribute selection. At the beginning of this process, data integration was carried out where three source files: Person, Household and Living Quarters. These were joined into a single dataset. Tables 2–4 show 40 attributes from person source file, 39 attributes from household source file and 17 attributes from living quarters source file. From a total of 96 attributes, repeated attributes were removed, leaving 84 attributes. Afterwards, two attributes were generated: salary and total household income based on occupation. These attributes mapped with Salaries & Wages Survey Report, Malaysia [32]. Then, the dataset was filtered to remove occupation from the category of unknown, unknown labor force status and unclassified. Non-B40 group and non-citizen were also filtered out from this study. Subsequently, data cleaning was done to fill in the missing values before the data transformation process takes place. Upon examination, there are 2,097 missing values from 2 attributes, namely, Country of Birth and Coding state/Country. The missing values for Country of Birth are replaced with the value ‘99’ which is ‘Malaysian Citizen’ while for the State/Country Code attribute, the missing values are replaced with the same values in State attribute. The operator called “Replace Missing Value” and it is used to replace every missing value with the specified values. In data transformation, a nominal attribute called age group was transformed into numeric attribute as there is a requirement for distance calculation in the clustering process. This process is performed by an operator called ‘Nominal to Numerical’ using unique integer coding type in Rapidminer. On top of that, normalization was performed using the Z-transformation method. It is important to note that normalization can ensure that the distance measure gives equal weight to each variable.

**Table 2:** A set of attributes from person source file.

No	Attributes	Description	Data type
1	Household ID	ID number for household	string
2	Living Quarter ID	ID number for living quarters	string
3	Household Member ID	ID number for household member	string
4	State	States in Malaysia	string
5	District	Administrative Districts	string
6	Strata	Urban/ Rural	num
7	Living Quarter No	Living Quarter Number	string
8	Household No	Household Number	string
9	Person No	Number of Household Member	string
10	Relationship	Relationship to Head of Household	string
11	Gender	Gender	numerical
12	Age	Age	string
13	Age Group	Age (5 year group)	string
14	Marital Status	Marital Status	numerical
15	Ethnic Group	Ethnic Group	numerical
16	Birthplace	Birthplace	numerical
17	State of birth	State of birth in Malaysia	string
18	Country of birth	Country of birth	string
19	Citizenship	Residence Status	numerical
20	Country of Citizenship	Country of citizenship	string
21	Place of Residence 5 Years Ago	Usual Place of Residence 5 Years Ago	numerical
22	Coding State/Country	State/Country Code	string
23	Coding District	District Code	string
24	Read and Write	Refers to literacy	numerical
25	Use Computer	Refers to computer literacy	numerical
26	Ever Been to School	Ever Been to School/Polytechnic/College/University	numerical
27	Highest Education	Highest Level of Education	numerical
28	Highest Certificate	Highest Certificate/Diploma/Degree	numerical
29	Work during the last 7 days	Work for at least 1 hour during the last 7 days	numerical
30	Work to return to	persons who did not work during the reference week but had a job, farm, enterprise or other family enterprise to return to	numerical
31	Look for work during the last 7 days	Look for work during the last 7 days	numerical
32	Reason for not seeking work	Reason for not seeking work	string
33	Occupation (1 Digit)	Major group for occupation	numerical
34	Industry (1 Digit)	Major group for Industry	numerical
35	Occupation (3 Digit)	Minor group for Occupation	string
36	Industry (3 Digit)	Minor group for Industry	string
36	Occupation Status	Employment status	numerical
38	Religion	Religion	string
39	Migration Status	5 Year of Migration Status	string
40	Labour Force Status	Labour Force Status	numerical

**Table 3:** A set of attributes from household source file.

No	Attributes	Description	Data type
1	Household ID	ID number for household	numerical
2	Living Quarter ID	ID number for living quarters	numerical
3	State	States in Malaysia	numerical
4	District	Administrative District	numerical
5	Strata	Urban/ Rural	numerical
6	Living Quarter No	Living Quarter Number	numerical
7	Household No	Household Number	numerical
8	1 Motor Car	Owned 1 Motor Car	numerical
9	2 Motor Car	Owned 2 Motor Car	numerical
10	3 or More Motor Car	Owned 3 or more Motor Car	numerical
11	1 Motorcycle	Owned 1 Motorcycle	numerical
12	2 or more Motorcycle	Owned 2 or more Motorcycle	numerical
13	Bicycle	Owned Bicycle	numerical
14	Air-conditioner	Owned Air-conditioner	numerical
15	Washing Machine	Owned Washing Machine	numerical
16	Refrigerator	Owned Refrigerator	numerical
17	Television	Owned Television	numerical
18	VCD/DVD Player	Owned VCD/DVD Player	numerical
19	Personal Computer	Owned Personal Computer	numerical
20	Laptop	Owned Laptop	numerical
21	Fixed Telephone Line	Owned Fixed Telephone Line	numerical
22	Mobile Phone	Owned Mobile Phone	numerical
23	Paid TV Channel	Owned Paid TV Channel	numerical
24	Digital Camera	Owned Digital Camera	numerical
25	Microwave Oven	Owned Microwave Oven	numerical
26	Internet Subscription	Subscribed to Internet	numerical
27	i-pod/PDA	Owned i-pod/PDA	numerical
28	Water Filter	Owned Water Filter	numerical
29	Radio/Hi-Fi	Owned Radio/Hi-Fi	numerical
30	None of the Items	Owned None of the Items	numerical
31	Ownership of Living Quarter	Ownership of Living Quarter	numerical
32	Ownership of other Living Quarter in Malaysia	Ownership of other Living Quarter in Malaysia	numerical
33	Rental Payment	Does the households paying rental for the living querter	numerical
34	Monthly Rental	Monthly rental payment amount	numerical
35	Type of Household	Type of Household	numerical
36	Composition of Household	Composition of Household	numerical
36	Total Male in Household	Total Male in Household	numerical
38	Total Female in Household	Total Female in Household	numerical
39	Total Persons in Household	Total Persons in Household	numerical

**Table 4:** A set of attributes from living quarters source file.

No	Attributes	Description	Data type
1	Living Quarter ID	ID number for living quarters	string
2	State	States in Malaysia	string
3	District	Administrative District	string
4	Strata	Urban/ Rural	numerical
5	Living Quarter No	Living Quarter Number	string
6	Type of Living Quarter	Type of living quarter	numerical
7	Living Quarter Housing Unit	Category of housing unit	string
8	Construction Material of Outer Walls	Construction Material of Outer Walls	numerical
9	Number of Rooms	Number of rooms in living quarter	string
10	Number of Bedrooms	Number of bedrooms in living quarter	string
11	Ownership Status	Ownership status of living quarter	numerical
12	Water Supply	Drinking water supply facility	numerical
13	Electricity Supply	Electricity supply facility	numerical
14	Toilet Facility	Toilet facility	numerical
15	Garbage Collection	Garbage collection facility	numerical
16	Total Persons in Living Quarter	Total persons in living quarter	numerical
17	Total Households in Living Quarter	Total households in living quarter	numerical

There are four steps involved in attribute selection. First, we delete useless attributes by using an operator called “Remove Useless Attributes” where the process identified attributes containing the same values for all the records. Second, we used “Remove Correlated Attributes” where it detects pairs of attributes that are strongly related to each other based on the correlation values specified. Third, we removed the non-significant ones, which is the id-like-attributes. Feature Selection methods can be classified into two major groups, which are supervised and unsupervised. In supervised feature selection methods, the features are chosen based on their association with the class label. It selects features with strong relevance to the class label. On the other hand, unsupervised feature selection methods evaluate the feature relevance by exploring the data structures with unsupervised learning techniques. In this study, an operator called ‘Unsupervised Feature Selection’ was used to select important attributes from a total of 65 attributes. Unsupervised Feature Selection technique uses K-Means algorithm to find the most important features. Table 5 provides a list of the 23 selected attributes after the selection process.

**Table 5:** A set of attributes after unsupervised feature selection.

No	Attributes	No	Attributes
1	Birthplace	13	Radio/Hi-Fi
2	Construction Material of Outer Walls	14	Reason for Not Seeking Work
3	Ever Been to School	15	Read and Write
4	Gender	16	Refrigerator
5	Highest Certificate	17	Strata
6	Highest Education	18	Toilet Facility
7	iPod/PDA	19	Type of Household
8	None of the Items	20	VCD/DVD Player
9	Occupation	21	Washing Machine
10	Occupation Status	22	Water Filter
11	Paid TV Channel	23	Work during the last seven days
12	Personal Computer		

### K-means algorithm

K-Means algorithm is one of the most popular and widely used clustering algorithms. It is a clustering method where n objects *o*_*1*_,…, *o*_*n*_ are clustered into a number of cluster *k* C_1_,…, *C*_*k*_. The initial group will be repeated several times by clustering each object to the nearest centroid point, and the centroid point will be recalculated until no further changes occur. The purpose of the optimization criteria in the clustering process are to minimize the sum of variances (Sum of Squared Errors) *E* between the objects in the cluster with the *cen*_1_,…, *cen*_k_ points such as Eq (1).


$$E=\sum_{i=1}^{k}\sum_{o\in C_{i}}{dist(o,{cen}_{i})}^{2}$$ (1)

In the K-Means algorithm, the distance is calculated between each data point and each centroid. The centroid is selected for each data point based on the minimum distance. Thus, distance plays an important role in the clustering process. Calculation of distance between these two points can be carried out using several techniques. Four distance measures are compared in this study namely Euclidean Distance, Correlation Similarity, Cosine Similarity and Dice Similarity. The Euclidean distance between two points is calculated based on Eq (2), where k is the number of dimensions, *aj* and *bj* are vectors: *a* = (*a1*, *a2*,…, *ak*), *b* = (*b1*, *b2*,…, *bk*). The dimensions used need to be transformed to be within the same scale, which is also known as normalization [33].


$$\sum_{j=1}^{k}{{(a}_{j}-b_{j})}^{2}$$ (2)

Correlation Similarity is calculated as the correlation between two attribute vector points. Given the data matrix X (*m x n*) where *m* (1 *x n*) line vectors *x1*, *x2*,…, *xm*, the correlation distance between *x*_*δ*_ and *x*_*t*_ vectors is defined as Eq (3) [31].


$$1-\frac{(x_{\delta }-{\bar{x}}_{\delta }){\left(x_{t}-{\bar{x}}_{t}\right)}^{\prime }}{\sqrt{\left(x_{\delta }-{\bar{x}}_{\delta })(x_{\delta }-{\bar{x}}_{\delta }\right)}\prime \sqrt{(x_{t}-{\bar{x}}_{t}){\left(x_{t}-{\bar{x}}_{t}\right)}^{\prime }}}$$ (3)

Cosine similarity is measured based on the cosine angle between two points of the attribute vector. Given a data matrix *X* (*m* * *n*) where m (1 * *n*) is the vector of the lines *x*_*1*_, *x*_*2*_,…, *x*_*m*_, the cosine distance between the vector *x_δ_* and *x_t_* is defined as Eq (4) [33].


$$d_{\delta t}=1-\frac{x_{\delta }{x\prime }_{t}}{\sqrt{\left(x_{\delta }{x\prime }_{\delta })(x_{t}{x\prime }_{t}\right)}}$$ (4)

Dice similarity used in this study is dice similarity for numerical values in the input set. For the distance measure, the y(*i*,*j*) is the value of the *j*^*th*^ attribute of the *i*^*th*^ instance. Hence y (1,3)—y(2,3) is the difference of the values of the third attribute of the first and second instance. The similarity is calculated using Eq (5), where Y_1_ Y_2_ is the sum over product of values which is sum (*j* = 1) y(1,*j*) * y(2,*j*). Y_1_ is the sum over values of the first instance which is sum (*j* = 1) y(1,*j*), while Y_2_ is the sum over values of the second instance which is sum (*j* = 1) y(2,*j*) This types of similarity measured is offered in Rapidminer tools for K-Means clustering algorithm [34].


$$2(Y_{1}Y_{2})/(Y_{1}+Y_{2})$$ (5)

The evaluation of clustering results, also called cluster validation, is a process in which the accuracy or quality of the results obtained from the cluster is measured. Two main methods for measuring the quality of cluster results are internal and external validation. The evaluation of external validation is based on the comparison of cluster results with the unused data in the clustering process. Unused data is the data which contains the class labels. The cluster results are considered good if the comparison results are similar. Some of the measurement methods in external validation are Jaccard Index, Rand-Index and F-measure [35]. Whereas, internal validation provides a good score to the algorithms that produce high similarity within a cluster and low similarity between clusters. Davies Bouldin Index [36], Dunn Index [37] and Silhouette Index [38] are the popular methods for internal validation measure. There are also some new clustering validation indices proposed such as clustering validation index based on nearest neighbors (CVVN index) [39], Local Cores-based Cluster Validity (LCCV index) [40] and Absolute Cluster Validity index [41]. For this study, three internal validations implemented, which were Davies Bouldin, Average within Centroid Distance and Sum of Squares.

#### Davies Bouldin

The Davies Bouldin (DB) metric measures the variation between points within the cluster (intra-cluster) and the distance between clusters (inter-cluster). In each cluster, this metric determines which other group has the highest ratio between the average intra-cluster distance of points in two clusters to the distance between clusters. After obtaining the maximum value, it will be averaged for all clusters. Low values are obtained if the distance within cluster is compact and the distance between cluster is far away. This measurement metric can provide clear clues for a good cluster [42]. This metric is defined as Eq (6):

$$DB(U)=\frac{1}{c}\sum_{i=1}^{c}\underset{j\neq 1}{\max}(\left\{\frac{\Delta (x_{i})+\Delta (x_{j})}{\delta (x_{i},x_{j})}\right\}$$ (6)

where δ(*x*_*i*_, *x*_*j*_) is the distance between cluster, *x*_*i*_, and *x*_*j*_, Δ*x*_*i*_, Δ*x*_*j*_ represent the distance between the points within cluster *x*_*i*_, and *x*_*j*_ is the centroid for cluster *x*_*i*_ and *c* are the numbers of partition *U* cluster.

#### Average within centroid distance

Average within Centroid Distance (AWCD) metric is measured by calculating the average distance per point from a centroid point within a cluster. The centroid distance between cluster *A* and *B* is the distance between centroid (*A*) and centroid (*B*). Average distance (*dist*) is calculated by finding the average in pairs between points within a cluster. In other words, for each point *a*_*i*_ in cluster *A*, the average distance is calculated *dist*(*a*_*i*_,*b*_*1*_), *dist*(*a*_*i*_,*b*_*2*_), … *dist*(*a*_*i*_,*b*_*n*_) and average them all. The more compact a cluster is, the lower the average value. This is because as the number of clusters increases, the average distance decreases naturally. This makes these measurement metrics difficult to interpret [42].

#### Sum of squares

Sum of Squares (SS) metric divides the number of data points in a group by the number of data points in each cluster. This is called squared, and the values of all the clusters are summed. This evaluation metric shows that a good cluster can change according to the starting parameters used to form the cluster. If the size of the scale decreases slowly with increasing numbers of clusters, it indicates that there is a large stable cluster that is still intact. Eq (7) shows the calculation of SS evaluation metrics [43]:

$$SS=\sum_{i=1}^{k}\sum_{x\in S_{i}}{\left\|x_{-}\mu_{i}\right\|}^{2}$$ (7)

where *S*_*i*_ represents the set of clusters (*S*_*1*_, …, *S*_*k*_) with a midpoint (*μ*_*1*_, …, *μ*_*k*_), *k* represents the number of clusters and *x* represents the data set.

## Result and analysis

### Determining the best distance

A series of experiment was run with *k* values ranging from 2 to 15 with four different distance measures, namely Euclidean Distance (ED), Correlation Similarity (CrS), Cosine Similarity (CS) and Dice Similarity (DS). Performance is measured based on DB, AWCD and SS evaluation metrics. Low values are representative of a good cluster with a particular distance measure. Table 6 shows the clustering performance based on three evaluation metrics (i.e., DB, AWCD and SS) for all *k* values starting from 2 to 15 using four different distance techniques (i.e., ED, CrS, CS and DS). The average DB values for the ED, CrS, CS and DS techniques were 1.78, 2.20, 2.19 and 5.91, respectively. As shown in Table 6, the DB recorded four infinity values when using the CrS technique at the *k* = 2, 6, 11and 14. At the same time, the DS technique recorded ten infinity values at the k = 3, 5, 7, 9,10,11,12,13,14 and 15. This indicates poor clustering quality results are produced when using the CrS and DS techniques based on the DB metric. Furthermore, as shown in Table 6, the average AWCD values were 13.98, 15.47, 14.96 and 23.87 for ED, CrS, CS and DS techniques, respectively. This shows that ED is the best distance technique compared to others (i.e., CrS, CS and DS) based on the average of DB and AWCD values. On the other hand, the CS technique is shown to outperform other distance techniques when using SS. This is based on the average values for all distance calculation techniques, which are 0.25, 0.23, 0.18 and 0.22 for ED, CrS, CS, and DS techniques.

**Table 6:** Clustering performance based on Davies Bouldin, average within centroid distance and sum of squares for *k = 2 to 15* based on Euclidean distance, correlation similarity, cosine similarity and dice similarity.

*k*	Davies Bouldin (DB)				Average within Centroid Distance (AWCD)				Sum of Squares (SS)			
	Distance Techniques				Distance Techniques				Distance Techniques			
	ED	CrS	CS	DS	ED	CrS	CS	DS	ED	CrS	CS	DS
2	2.00	∞	2.39	5.64	19.85	23.00	20.00	23.39	0.65	1.00	0.53	0.50
3	1.90	2.64	2.54	∞	18.19	19.08	18.73	23.39	0.50	0.36	0.33	0.50
4	2.03	2.34	2.50	6.58	17.25	17.40	17.37	23.40	0.41	0.28	0.26	0.27
5	2.08	2.43	2.23	∞	16.02	16.75	16.26	23.28	0.28	0.21	0.23	0.25
6	1.78	∞	2.21	5.80	15.05	16.34	15.63	24.08	0.29	0.23	0.18	0.18
7	1.86	2.15	2.18	∞	14.36	15.19	15.22	23.76	0.24	0.20	0.15	0.25
8	1.60	2.23	2.16	5.64	13.41	15.10	14.41	24.21	0.24	0.15	0.13	0.15
9	1.80	2.19	2.22	∞	12.97	14.31	14.20	24.64	0.16	0.13	0.11	0.15
10	1.56	2.12	2.15	∞	12.18	14.49	13.84	23.98	0.19	0.13	0.10	0.13
11	1.70	∞	2.17	∞	12.19	14.04	13.73	23.59	0.15	0.12	0.10	0.21
12	1.63	2.19	2.10	∞	11.21	13.47	13.27	23.95	0.12	0.11	0.09	0.14
13	1.68	1.82	2.03	∞	11.13	12.45	12.48	24.51	0.12	0.10	0.08	0.12
14	1.65	∞	1.97	∞	11.03	12.90	12.18	24.02	0.11	0.11	0.08	0.14
15	1.63	1.91	1.90	∞	10.83	12.06	12.08	23.97	0.10	0.09	0.08	0.11
Average	1.78	2.20	2.19	5.91	13.98	15.47	14.96	23.87	0.25	0.23	0.18	0.22

Moreover, to select the best distance technique, their performance is measured based on DB, AWCD and SS evaluation metrics. Table 7 demonstrates the average clustering performance for each distance measured. The ED technique recorded the best performance results based on the lowest DB and AWCD values. Meanwhile, the CS is the best distance technique that can be used to produce a quality clustering model based on SS value. Moreover, CrS shows moderate performance, and DS reveals a poor clustering performance. The performance results recorded in Table 7 are ranked from 1 to 4 for each evaluation metric to select the best distance measure.

**Table 7:** Comparison of average clustering performance based on distance measure.

Distance Measure	DB	AWCD	SS
Euclidean Distance	1.78	13.98	0.25
Correlation Similarity	2.20	15.47	0.23
Cosine Similarity	2.19	14.96	0.18
Dice Similarity	5.91	23.87	0.22

Table 8 shows the list of ranks for each distance measure based on the average values of DB, AWCD and SS metrics. The distance technique with the average value for each evaluation metric is recorded. From these values, the rank for each distance technique was noted for the purpose of identifying the performance of the distance technique. Thus, in these studies, the distance technique subjects are ranked (1 to *n*), so the rank value is from 1 to 4. For example, DB produces the lowest average value for ED. Therefore, ED was ranked as number 1, and the DS technique, with the highest average DB value, will have rank number 3. The final two columns on the right in Table 8 are the mean of the rank and rank position obtained of all evaluation metrics for each distance measure. This produces a listed rank position for each distance measure. Overall, the resulting ranking of the four distance measures is:

Cosine Similarity > Euclidean Distance > Correlation Similarity > Dice Similarity

It is shown that the Cosine Similarity is the best distance technique based on the lowest score obtained.

**Table 8:** Final score ranking to select the best distance measure.

Distance Measure	DB	AWCD	SS	Mean Rank	Ranking Position
Euclidean Distance	1	1	4	2.00	2
Correlation Similarity	3	3	3	3.00	3
Cosine Similarity	2	2	1	1.67	1
Dice Similarity	4	4	2	3.33	4

### Determining the best k value

K-Means algorithm is an easy clustering algorithm. However, it requires the parameter *k* as the input to the clustering process. Variable *k* is an important parameter in determining the quality of a cluster. Therefore, this study will determine the best *k* value for the clustering model. The performance graph for the model is plotted based on Cosine Similarity measure.

**Fig 2:**
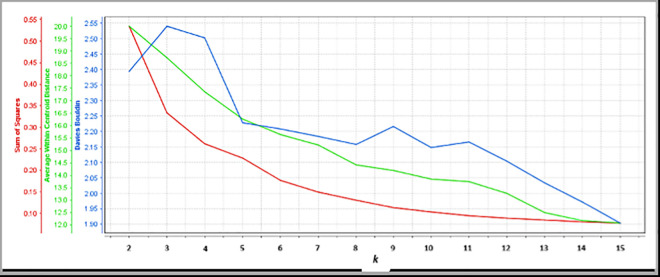
Cluster performance plot.

Fig 2 shows the performance plotting of the clustering model from k = 2 up to k = 15. According to Davies Bouldin (DB) measure, a low DB value indicates that the clusters are tight, and each cluster is well separated. Based on the DB measure, the lowest value is recorded by k = 15. Based on the Average within Centroid Distance (AWCD) plots, the AWCD values seem to flatten at k = 8. This indicates that an increasing number of clusters does not significantly affect the quality of the clusters [42]. Based on the Sum of Squares (SS) measure, the SS value drops dramatically until k = 8 before it begins to flatten. Therefore, based on DB measure and taking into account the ACWD and SS measure, it can be concluded that k = 8 with DB = 2.157 is the best k value for this model.

### Clustering analysis

The analysis and interpretation of cluster results are one of the most important activities in clustering. Each cluster needs to be explored and analyzed to get its characteristics and differences. In this study, the analysis and interpretation of each cluster will determine the indicators and dimensions for multidimensional poverty among B40 group. The analysis of each cluster was done by looking at the cluster size, centroid chart, scatter plot analysis, heat map analysis, and descriptive statistics method.

#### Cluster size analysis

As shown in Table 9, eight clusters are derived from the clustering model. Cluster 0 and 2 constitute the largest group comprising 16% each. Both clusters had an average distance with the lowest average centroid distance, indicating more compact clusters. Whereas, the smallest cluster is Cluster 3, making up 9% of the entire cluster. On the other hand, the Average within Centroid Distance (AWCD) returned a lower value for Cluster 2 at 9.064, which indicates that Cluster 2 is the most compact cluster than the other clusters.

**Table 9:** Size of cluster.

Cluster	No of Individual	Cluster Size (%)	Average within Centroid Distance
0	46,430	16	12.347
1	31,076	11	15.329
2	45,459	16	9.064
3	26,540	9	20.192
4	28,437	10	16.909
5	30,950	11	14.972
6	42,710	15	13.028
7	35,496	12	18.218
**Total**	287,098	100	
**Average**			14.437

#### Centroid chart analysis

**Fig 3:**
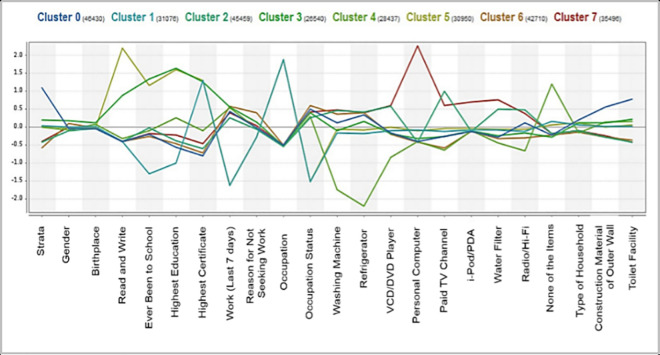
Centroid chart.

The Centroid Chart, as shown in Fig 3, is a graphical representation of centroid value in a parallel chart. It represents the mean value of centroid point for the given attribute for each of the cluster. The centroid value is a normalized value; therefore, the mean value for each attribute is equal to 0. The centroid value, which is far above and below the mean value can easily be noticed through this chart, which indicates a distinguishing characteristic for the respective cluster. For instance, for Cluster 7, the centroid value for personal computer attribute is 2.27, which is far above the mean value and is the highest value as compared to other clusters. Thus, the ‘personal computer’ is one of the most important characteristics of Cluster 7.

Nevertheless, this form of analysis offers minimal insights; thus, the indicators and dimensions for multidimensional poverty cannot be specified at this point. Therefore, we proceed to the next analysis called Scatter Plot analysis.

#### Scatter plot analysis

**Fig 4:**
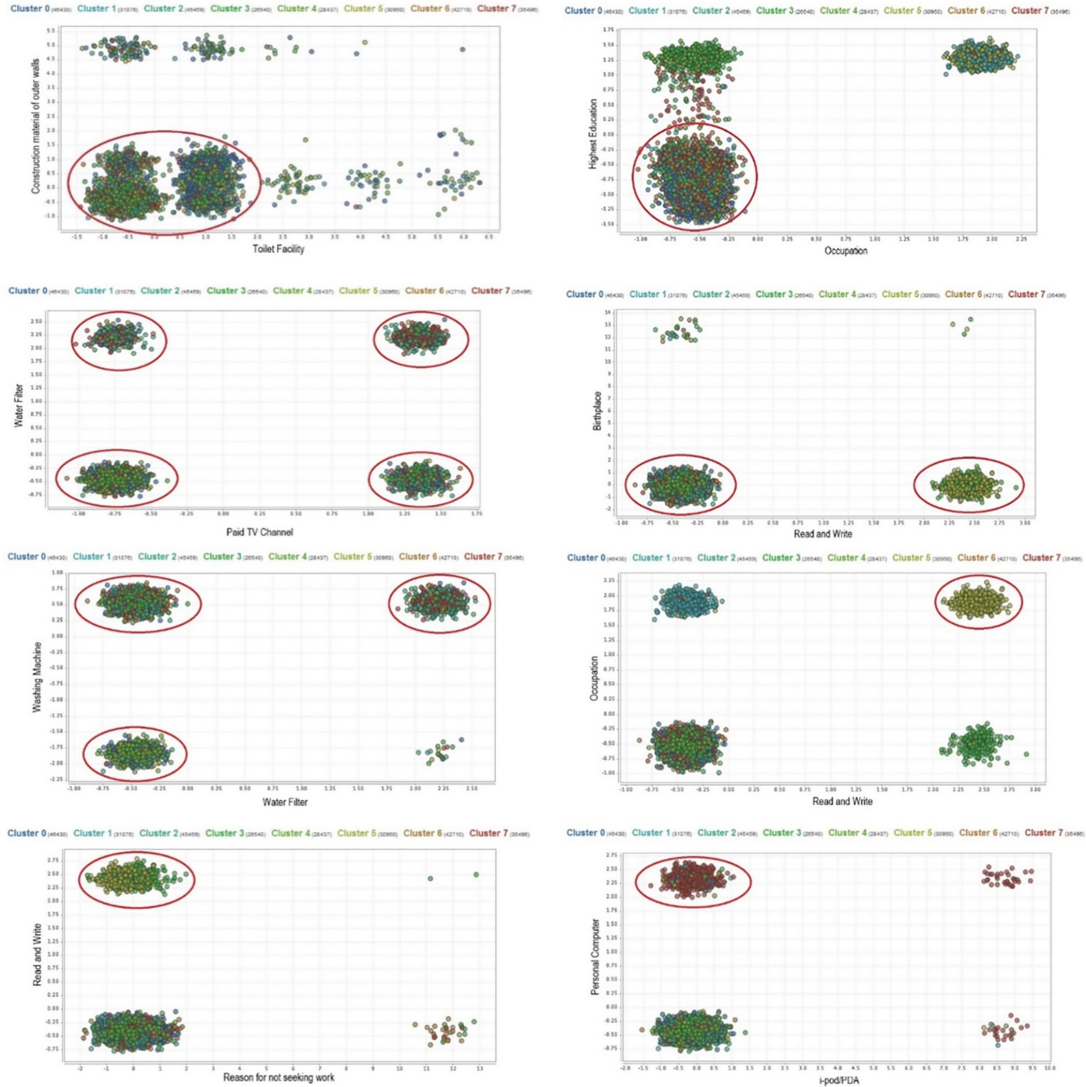
Scatter plot of (a) Cluster 0; (b) Cluster 1; (c) Cluster 2; (d) Cluster 3; (e) Cluster 4; (f) Cluster 5; (g) Cluster 6; (h) Cluster 7.

Scatter plots are another way of analyzing cluster characteristics graphically. It is very useful in visually positioning the cluster based on two key attributes of each cluster. It indicates the relationship or correlation between these two attributes. In light of this scatter plot analysis, 12 attributes have been selected as key indicators in defining each cluster as depicted in Fig 4A–4h.

As seen in Fig 4(A), Cluster 0 shows a relationship between toilet facilities and construction material of outer walls attribute. These group of people probably experienced a low living standard. There is a big and compact cluster in Cluster 1 that shows the strong correlation between the highest certificate and occupation attribute, as shown in Fig 4(B). Based on the plotting, most of the individuals in this cluster are not working and do not have any certification. Whereas, scatter plot for Cluster 2, as shown in Fig 4(C), depicts the correlation between paid TV channel and water filter attribute. Fig 4(D) shows plotting for Cluster 3, which reflects the remaining population between B40 group who are able and unable to read and write. Meanwhile, for cluster 4, as shown in Fig 4(E), a similar proportion can be seen between people of B40 group who owned a washing machine and a water filter. Plotting in Cluster 5 presents a strong correlation between the attribute of occupation and the capability to read and write, as shown in Fig 4(F). On the other hand, Cluster 6 revealed that majority people from this group are not working based on the reason of ‘not seeking work’ attribute, as shown in Fig 4(G). But majority people in this group have the ability to read and write. They might be the children or spouse of the head of the household. Lastly, Cluster 7 exposed that majority of B40 individual from this group owned a personal computer, and some of them owned an iPod/PDA, as shown in Fig 4(H). This pattern of plotting indicates a good standard of living of people in the cluster.

#### Heat map analysis

**Fig 5:**
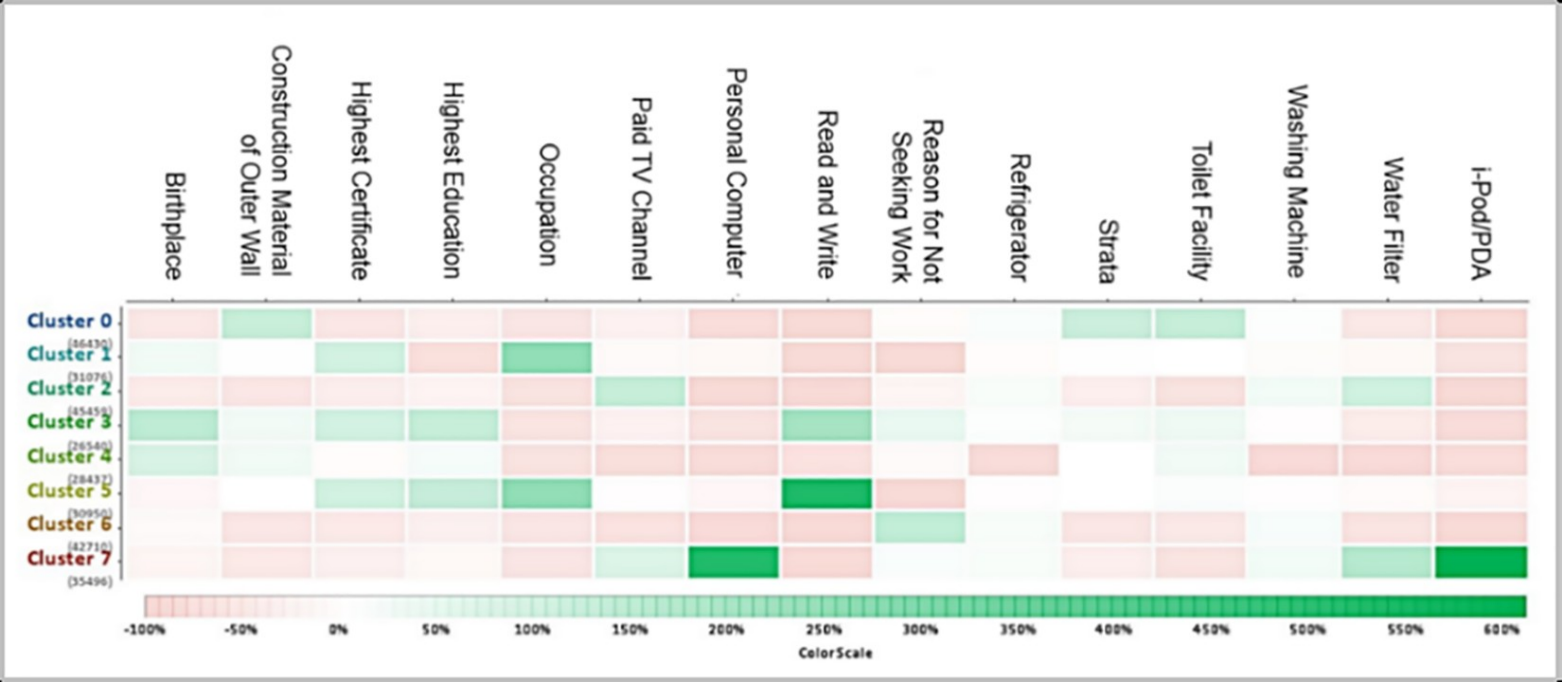
List of important attributes for B40 clustering model from heat map analysis.

As compared to the scatter plot, the heat map analysis is able to reveal more than two important attributes for each cluster, whereby these attributes have a strong correlation. Heat map analysis is ideal for large-scale data visualization. The color scale shows the importance of the attributes where light green indicates an attribute with a high centroid value, and pink indicates an attribute with a low centroid value. From a total of 23 attributes, 15 attributes have been selected from heat map analysis and labelled as important attributes in forming the clusters. These are strata, birthplace, read and write, highest education, highest certificate, toilet facility, construction material of outer walls, paid TV channel, water filter, refrigerator, washing machine, occupation, reason for not seeking work, personal computer and iPod/PDA, as shown in Fig 5. There are three other extra attributes as compared to the scatter plot analysis which are highest education, refrigerator and strata. Each of these 15 attributes will be further analyzed in the next analysis called Descriptive Statistics Methods to identify the multidimensional indicators and dimensions in the context of B40 group in this study.

#### Descriptive statistics method

Based on the most important attributes identified in the previous analysis, descriptive statistics method is employed for further analysis in understanding the data within each cluster in order to identify the most relevant indicators and dimensions for multidimensional poverty. Descriptive statistics is a method that gives an overview or summary of a data through numerical calculations, graphs or tables [44]. Descriptive statistics on cluster results can provide a detailed picture on how similar the attributes are in the cluster [45].

Indicators and dimensions are two most important components of MPI in defining poverty. Indicators should capture the deprivation experienced, while dimensions are the grouping of indicators [46, 47]. There are many methods for selecting MPI indicators and dimensions. The most relevant MPI indicators and dimensions for the B40 group will be specified in this analysis. The naming and grouping of indicators identified in this analysis are referring to a discussion on guideline provided in [48]. For that reason, the values for each attribute discussed before need to be denormalized to see the actual values in order to achieve a meaningful interpretation result. Table 10 provides descriptive statistics for the B40 group clustering model. The grey color columns indicate the distinguishing characteristics for each cluster based on the statistics obtained.

**Table 10:** Descriptive statistics for B40 clustering model.

Attributes	Cluster 0	Cluster 1	Cluster 2	Cluster 3	Cluster 4	Cluster 5	Cluster 6	Cluster 7
Read and Write	100% Yes	100% Yes	100% Yes	55% Yes	97% Yes	92% No	100% Yes	99% Yes
				45% No				
Highest Education	35% Primary	66% Primary	70% Secondary	61% No education	26% Not applicable	99% No education	64% Secondary	62% Secondary
	61% Secondary	34% Pre-School	19% Primary	34% Not applicable	38% Secondary		26% Primary	17% Primary
					19% Primary			
Highest Certificate	31% UPSR	100% No certificate	48% SPM/STPM	61% No certificate	40% Not applicable	99% No certificate	39% SPM/STPM	42% SPM/STPM
	30% SPM/STPM		19% UPSR	34% Not applicable	25% SPM/STPM		24% UPSR	18% UPSR
	19% PMR/SRP		17% PMR/SRP		17% UPSR			
Strata	79% Rural	69% Urban	89% Urban	61% Urban	68% Urban	69% Urban	97% Urban	88% Urban
	21% Urban	31% Rural	11% Rural	39% Rural	32% Rural	31% Rural		
Birthplace	99% Malaysia	99% Malaysia	99% Malaysia	98% Malaysia	99% Malaysia	99% Malaysia	99% Malaysia	99% Malaysia
Toilet Facility	71% Pour Flush	69% Flush system	91% Flush system	59% Flush system	64% Flush system	68% Flush system	87% Flush system	90% Flush system
		28% Pour Flush		37% Pour Flush	29% Pour Flush	29% Pour Flush	13% Pour Flush	
Construction Material of Outer Walls	35% Brick	70% Brick	88% Brick	58% Brick	64% Brick	69% Brick	86% Brick	89% Brick
	32% Brick and Plank	15% Brick and Plank	7% Brick and Plank	22% Plank	22% Plank	16% Plank	7% Plank	7% Brick and Plank
	25% Plank	11% Plank		16% Brick and Plank	7% Brick and Plank	11% Brick and Plank		
Paid TV Channel	78% No	70% No	84% Yes	77% No	96% No	66% No	92% No	65% Yes
						34% Yes		35% No
Water Filter	94% No	86% No	64% No	92% No	99% No	85% No	95% No	54% Yes
			36% Yes					46% No
Refrigerator	98% Yes	78% Yes	99% Yes	91% Yes	94% No	82% Yes	99% Yes	99% Yes
Washing Machine	82% Yes	71% Yes	97% Yes	73% Yes	96% No	75% Yes	93% Yes	98% Yes
Occupation	67% No	100% No (below age 10 years)	52% No	54% No	48% No	99% No (below age 10 years)	76% No	64% No
Reason for Not Seeking Work	34% Still schooling	100% Not applicable	48% Not applicable	46% Not applicable	52% Not applicable	100% Not applicable	38% Still schooling	37% Not applicable
	34% Not applicable		23% Still schooling	28% Retired	33% Still schooling		25% Not applicable	33% Still schooling
	18% Housewife		17% Housewife	18% Housewife			20% Housewife	18% Housewife
Personal Computer	99% No	87% No	100% No	96% No	99% No	88% No	99% No	98% Yes
iPod/PDA	100% No	99% No	100% No	99% No	99% No	99% No	100% No	91% No 9% Yes
CLUSTER SIZE (individuals)	46,430	31,076	45,459	26,540	28,437	30,950	42,710	35,496

*Attributes analysis in defining multidimensional poverty indicators and dimensions*. Reading and writing is a basic literacy skill which indicates the ability of a person to read and write. By referring to Table 10; this attribute was a distinct characteristic for Cluster 3 and 5, which 45% and 92% of individuals from Cluster 3 and 5, respectively, were not able to read and write. Highest education refers to the highest level of education attained by a person which includes pre-primary, primary, secondary, pre-university and tertiary. Table 10 reveals that this attribute was recognized as an important variable in distinguishing Clusters 3 and 5 in which 61% and 99% of people from Cluster 3 and 5 had no education. While the ‘not applicable’ classification in Cluster 3 refers to individuals who are too young or never attended school. A similar pattern can be seen for the highest certificate attribute with an additional cluster, which is Cluster 1. The ‘highest education level’ attribute has been observed in a large percentage in Cluster 1, which clearly indicates that this cluster consists of minors which 100% of people attained primary and pre-school education. Education is one of the dimensions of global MPI and is closely related to poverty. Thus, literacy indicator proposed in this study consists of reading and writing attribute while the highest level and grade indicator are introduced, which consist of highest education and highest certificate attributes. These two indicators are grouped under the education dimension to measure the education level among the B40 group.

Strata attribute refers to a person’s living environment, urban or rural. Table 10 shows peoples from a rural area dominated cluster 0, while Cluster 1, 3, 4, 5 have more than 30% individuals from rural areas. This attribute has been observed to have a correlation with toilet facility and construction material of outer walls attributes. A greater percentage of people from these clusters are using the pour-flush toilet and living in a house made of plank or a combination of brick and plank. This proved that people living in rural area have a lower living standard as compared to urban people. Although strata is one of the important variables in cluster formation, however, this attribute is considered as a demographic variable. Another demographic attribute found is the birthplace attribute. Thus, both attributes are not selected as multidimensional poverty indicators.

Five types of toilet facilities were listed in Malaysia, namely the flush system, pour-flush, pit, enclosed space over water and none. The Malaysian MPI used this attribute as one of the indicators to measure poverty which defines households without flush system as the cut-off for deprivation. However, global MPI used different terminology, which is sanitation with a different cut-off measure. Table 10 reveals Cluster 0 is the most deprived when it comes to the toilet facility with 71% using pour flush toilet, followed by the other 4 clusters: Cluster 1, 3, 4 and 5. Therefore, the toilet facility attribute is selected as an attribute of measure for sanitation indicator in this study.

The construction material of outer walls is another important attribute derived from the B40 clustering model. As seen in Table 10, there are 5 clusters, out of which less than 70% lived in houses made of brick. This attribute is one of the items defined by global MPI under housing indicator. Thus, the housing indicator is suggested in this study with construction material of outer walls as the measure attribute.

Paid TV channel attribute has been identified as a distinct characteristic for Cluster 2 and 7. A higher percentage of people were observed in these two clusters: 84% and 65% from Cluster 2 and 7, respectively could afford to subscribe to the service while most people in Cluster 4 and 6 cannot. This indicates a good standard of living for both clusters. In total, only 36% of the total dataset have access to this service. This attribute is suggested to be an attribute of measure for a new indicator called access to television service.

As it can be seen in Table 10, the water filter attribute is observed to be related to the paid TV channel attribute where people who are able to subscribe to the television service are also able to own a water filter. A total of 83% of the dataset does not own any water purification system at home. Safe drinking water is critical for public health, and water purification system can help to produce safe drinking water, especially for a rural area that did not get treated water supply. Hence, this attribute is also selected as one of the indicators. Refrigerator and washing machine are the two most common home appliances. Statistics, however, indicates that the majority of people from Cluster 4 are living without these two appliances. Therefore, these three home appliances: water filter, refrigerator and washing machine are chosen as measure attributes for assets indicator.

Occupation attribute refers to major groups of occupation in Malaysia based on the International Standard Classification of Occupations (ISCO-08). Occupation is the main source of income for most of the households in Malaysia. A total of 45% from the dataset of this study were categorized under outside labor force which means that they were unemployed, 22% were under ten years old who were the children of the head of the households, and the rest were employed people from various types of occupation. Table 10 indicates that Cluster 1 and 5 were the children of the head of the households, and Cluster 6 has the most significant unemployment percentage. ‘Reason for not seeking work’ attribute reveals about the unemployment percentage in occupation attribute, and hence both attributes are selected to measure multidimensional poverty under work indicator.

Both personal computer and iPod/PDA are other assets of B40 people, and both are distinguished features for Cluster 7. Table 10 illustrates 98% of people in Cluster 7 owned a personal computer, and 9% of this cluster owned an iPod/PDA. This indicates that this cluster is relatively good in standard of living due to their ability to own technology assets. Considering the importance of technology as the key growth engine for the emerging and developing country like Malaysia, these two attributes are selected to be measure attributes under assets indicator.

The analysis discussed above results in new multidimensional poverty measure for B40 group includes three dimensions: Education, Living Standards and Employment being broken down by seven indicators namely literacy, highest education level and grade, sanitation, housing, access to television services, assets and work with 13 measure attributes namely Read and Write, Highest Education, Highest Certificate, Toilet Facility, Construction Material of Outer Walls, Paid TV Channel, Water Filter, Refrigerator, Washing Machine, Personal Computer, iPod/PDA, Occupation, Reason for Not Seeking Work as presented in Table 11. Whereas, Table 12 provides a comparison between global MPI, Malaysia MPI and MPI discovered in this study.

**Table 11:** Dimensions, indicators and measure attributes identified from the B40 clustering model.

DIMENSIONS	INDICATORS	MEASURE ATTRIBUTES
EDUCATION	Literacy	Read and Write
	Highest education level and grade	Highest Education
		Highest Certificate
LIVING STANDARDS	Sanitation	Toilet Facility
	Housing	Construction Material of Outer Walls
	Access to television services	Paid TV Channel
	Assets	Water Filter
		Refrigerator
		Washing Machine
		Personal Computer
		iPod/PDA
EMPLOYMENT	Work	Occupation
		Reason for Not Seeking Work

**Table 12:** Dimensions and indicators comparison.

MPI Dimensions and Indicators		
Global MPI (2018)	Malaysia MPI (2016)	This study (2020)
EDUCATION • Years of Schooling • School Attendance	EDUCATION • Years of schooling • School attendance	EDUCATION • Literacy • Highest education level and grade
HEALTH • Nutrition • Child Mortality	HEALTH • Access to health facility • Access to clean water supply	EMPLOYMENT • Work
LIVING STANDARDS • Cooking Fuel • Sanitation • Drinking Water • Electricity • Housing • Assets	LIVING STANDARDS • Conditions of Living Quarters • Number of Bedrooms • Toilet Facility • Garbage Collection Facility • Transportation • Access to Basic Communication Tools	LIVING STANDARDS • Sanitation • Housing • Assets ownership • Access to television services
	INCOME • Mean monthly household income	

Malaysia citizens are categorized into three different income groups, which are the Top 20 Percent (T20), Middle 40 Percent (M40), and Bottom 40 Percent (B40). The B40 group is further divided into three subgroups: lower-middle income, low-income, and poor. The success of the B40 clustering model in identifying B40’s new important indicators can help to improve the present MPI’s ability to detect the poor group. Additionally, it can also help to enhance poverty measurement based solely on income, namely the PLI. This can be seen by comparing the PLI method’s poverty measurement with the new MPI calculated using this data set. With PLI, the number of B40s in each sub-category is distributed in Table 13. The B40 group can be categorized as poor with 14%, low income at 50%, and low middle income at 36%.

**Table 13:** Distribution of B40 group based on 2016’s PLI.

Cluster	*n*	Poor	Low-income	Lower-middle income
		<RM 981	RM 981- RM 2614	>RM 2614
0	46430	4299	27127	15004
1	31076	4320	16819	9937
2	45459	3955	22275	19229
3	26540	6085	11418	9037
4	28437	6102	11970	10365
5	30950	3350	17027	10573
6	42710	7234	22081	13395
7	35496	3904	16203	15389
Grand total (*n*)	287098	39249	144920	102929
Percentage		14%	50%	36%

Eight sub-categories of B40 were discovered using the new MPI. According to verifications by poverty experts, Cluster 3 contains features of poverty that leads to the poor group. This is due to the fact that it has the smallest cluster size, which is 9% of the population, as shown in Table 9, the lowest average income as shown in Table 13, and possing the characteristics of poor people. As a result, Cluster 3 depicts the poor characteristics in this data set, as described in Table 14.

**Table 14:** Poor characteristic from Cluster 3.

Attributes	Cluster 3	Description
Read and Write	55% Yes	55% Poor Can Read and Write while 45% Cannot
	45% No	
Highest Education	61% No education	61% Poor does not have education while 34% not applicable
	34% Not applicable	
Highest Certificate	61% No certificate	61% poor do not have a certificate and 34% not applicable
	34% Not applicable	
Strata	61% Urban 39% Rural	61% of poor live in urban while 39% live in a rural area
Birthplace	98% Malaysia	98% poor was born in Malaysia
Toilet Facility	59% Flush system	59% of the poor using flush system and 37% using pour-flush toilet system
	37% Pour Flush	
Construction Material of Outer Walls	58% Brick	58% of the poor live in a brick house, 22% in plank house and 16% mixed house
	22% Plank	
	16% Brick and Plank	
Paid TV Channel	77% No	77% of the poor do not have paid tv channel
Water Filter	92% No	92% of the poor do not have a water filter
Refrigerator	91% Yes	91% of the poor have a refrigerator
Washing Machine	73% Yes	73% of poor have a washing machine
Occupation	54% No	54% of the poor are unemployed.
Reason for Not Seeking Work	46% Not applicable	28% of the poor do not look for a job because they have already retired, and 18% are housewives.
	28% Retired	
	18% Housewife	
Personal Computer	96% No	96% poor do not have a personal computer
iPod/PDA	99% No	99% poor people do not have PDA

This comparison shows that just 9% of the poor are detected utilizing the new MPI method, compared to 14% using the PLI approach. Although the MPI can identify a smaller number of poor people than the PLI, it can look at a variety of different characteristics simultaneously that lead to a person being classified as poor. As a result, any government assistance programme aimed at lifting these people out of poverty can be targeted more precisely.

## Conclusion

One of the focus areas in the Eleventh Malaysia Plan (11MP) is to elevate the B40 household group towards the middle-income society. Based on recent studies by the World Bank, Malaysia is expected to enter the high-income nation between 2024 and 2028. Thus, it is essential to clarify the B40 population through data-driven analytics to develop a comprehensive action plan by the government. Data analytic concerns the extraction of meaning, patterns and trends from varied and large volumes of data. Such data sets exist in many areas, and poverty eradication is no exception. Currently, the measurement of absolute poverty in Malaysia is known as the Poverty Line Income (PLI). PLI is an income approach in one dimension, specifically measuring the gross monthly household income. In order for the B40 group to be deserving to be in the middle-class income, a striking attempt to improve the condition of the people in the group must be properly taken. At present, the B40 group is identified by income status, when in reality, they are more vulnerable to deprivations defined by numerous poverty dimensions. Malaysia has also employed the customised MPI technique from OPHI to measure multi-dimensional poverty. However, the World Bank Group has criticized the adoption of such techniques with only a detection rate of 0.86% and has urged that the benchmark, or so-called deprivation cut-off level, be raised. Thus, a clustering model-based K-Means Algorithm with Cosine Similarity measure is developed to form clusters of B40 group as one of alternative method by using machine learning to identify most important poverty indicator and its deprivation cut-off level. The evaluation found *k* = 8 to be the best *k* value for the model. A series of clustering analysis was then conducted to identify the indicators associated with multidimensional poverty and dimensions for the B40 community in Malaysia. By employing the descriptive statistics method, three dimensions have been established: Education, Living Standards and Employment with seven indicators: literacy, highest education level and grade, sanitation, housing, access to television services, assets and work. Out of seven indicators identified, this study proposed six new Multidimensional Poverty Indicators namely literacy, highest education level and grade, housing, access to television services, assets (water filter, refrigerator, washing machine, personal computer, iPod/PDA) and work to be considered by the policymakers as a valuable addition to the current MPI to establish a more meaningful picture of the current poverty trend in Malaysia. Furthermore, this study has discovered Cluster 3 of the B40 group to contain the smallest cluster size of 9% relative to the population with the lowest average income and possessing the characteristics of poor people, which had been confirmed by poverty specialists.

A further in-depth study should be carried out in future to get the other important Multidimensional Poverty Indicators (MPI) components which are deprivation cut-offs and weights for each of the indicators specified. These components should be obtained for computation of MPI value as an absolute multidimensional poverty measurement. Furthermore, the algorithm used in the grouping model is K-Means. Many other algorithms can be studied and tested that may improve the clustering quality. The development of those algorithms could further enhance the attractiveness of the clustering approach to identify MPI for Bottom 40 group. In addition, by 2021, a new collection of census data will be published; that is Population and Housing Census 2020. This latest data could be applied in the future, which could offer the latest trends and more reliable research results.
